# DNA methylation age is elevated in breast tissue of healthy women

**DOI:** 10.1007/s10549-017-4218-4

**Published:** 2017-03-31

**Authors:** Mary E. Sehl, Jill E. Henry, Anna Maria Storniolo, Patricia A. Ganz, Steve Horvath

**Affiliations:** 1 Medicine, Hematology-Oncology, David Geffen School of Medicine, University of California Los Angeles, Los Angeles, CA 90095 USA; 2 Biomathematics, David Geffen School of Medicine, University of California Los Angeles, Los Angeles, CA 90095 USA; 3 Susan G. Komen Tissue Bank at the Indiana University Simon Cancer Center, Indianapolis, IN 46202 USA; 4 Health Policy and Management, Fielding School of Public Health, University of California Los Angeles, Los Angeles, CA 90095 USA; 5 Department of Human Genetics, David Geffen School of Medicine, Gonda Research Center, University of California Los Angeles, 695 Charles E. Young Drive South, Box 708822, Los Angeles, CA 90095-7088 USA; 6 Biostatistics, School of Public Health, University of California Los Angeles, Los Angeles, CA 90095 USA

**Keywords:** Breast cancer, Tissue aging, DNA methylation, Epigenetics, Biomarker of aging, Estrogen, Cell cycling

## Abstract

**Background:**

Limited evidence suggests that female breast tissue ages faster than other parts of the body according to an epigenetic biomarker of aging known as the “epigenetic clock.” However, it is unknown whether breast tissue samples from healthy women show a similar accelerated aging effect relative to other tissues, and what could drive this acceleration. The goal of this study is to validate our initial finding of advanced DNA methylation (DNAm) age in breast tissue, by directly comparing it to that of peripheral blood tissue from the same individuals, and to do a preliminary assessment of hormonal factors that could explain the difference.

**Methods:**

We utilized *n* = 80 breast and 80 matching blood tissue samples collected from 40 healthy female participants of the Susan G. Komen Tissue Bank at the Indiana University Simon Cancer Center who donated these samples at two time points spaced at least a year apart. DNA methylation levels (Illumina 450K platform) were used to estimate the DNAm age.

**Results:**

DNAm age was highly correlated with chronological age in both peripheral blood (*r* = 0.94, *p* < 0.0001) and breast tissues (*r* = 0.86, *p* < 0.0001). A measure of epigenetic age acceleration (age-adjusted DNAm Age) was substantially increased in breast relative to peripheral blood tissue (*p* = 1.6 × 10^−11^). The difference between DNAm age of breast and blood decreased with advancing chronologic age (*r* = −0.53, *p* = 4.4 × 10^−4^).

**Conclusions:**

Our data clearly demonstrate that female breast tissue has a higher epigenetic age than blood collected from the same subject. We also observe that the degree of elevation in breast diminishes with advancing age. Future larger studies will be needed to examine associations between epigenetic age acceleration and cumulative hormone exposure.

**Electronic supplementary material:**

The online version of this article (doi:10.1007/s10549-017-4218-4) contains supplementary material, which is available to authorized users.

## Introduction

We recently developed a multi-tissue age estimator (referred to as epigenetic clock) that accurately estimates chronological age across multiple cells and tissues based on DNA methylation data [1]. The resulting age estimate is referred to as “epigenetic age” or “DNA methylation age.” An increasing body of literature shows that the epigenetic age estimate captures aspects of the biological age of the underlying tissue. For example, the epigenetic age of blood is predictive of all-cause mortality after adjusting for chronological age and known risk factors of mortality [2–5]. The epigenetic clock method has shown that certain tissues exhibit age acceleration due to obesity [6], Alzheimer’s disease [7], Down syndrome [8], Parkinson’s disease [9], HIV [10, 11], Huntington’s disease [12], lifetime stress [13], and menopause [14].

This epigenetic aging measure lends itself to contrasting the biologic ages of different tissues of the human body [15]. In an earlier publication, we presented evidence that female breast tissue may be biologically older than other parts of the body, using data from 82 publicly available Illumina datasets from many different tissues [1]. However, the earlier analysis surrounding epigenetic aging effects in breast tissue had a methodological limitation, which was that it was mostly based on normal adjacent tissues from breast cancer patients. The only normal breast tissue dataset (*N* = 23) in that study was used in the training data, and there were two normal adjacent breast tissue datasets (*N* = 81 and *N* = 27) from breast cancer patients from TCGA used in the test data. Since cancer field effects and other hidden biases may have confounded our original analysis, the purpose of this study was to validate the accelerated aging effect of female breast tissue using matched specimens from the breast tissue and peripheral blood from healthy female volunteers. Further, we aimed to expand our original analysis by examining longitudinal changes in epigenetic age in breast compared with blood using matched samples within subject over time, and adding a preliminary assessment of the influence of menopausal status and reproductive factors on the epigenetic age acceleration in breast and blood tissue.

## Methods

### Study specimens

This study made use of specimens from the Susan. G. Komen Tissue Bank (KTB) at the Indiana University Simon Cancer Center funded by Susan G. Komen Foundation. This is a unique resource developed with the goal of understanding normal breast biology, to better understand the disruption that occurs during breast carcinogenesis, and to accelerate breast cancer prevention research. Participants in the KTB are healthy tissue donors without a history of breast cancer.

We requested samples from a subset of healthy female donors who had provided both peripheral blood and breast tissue at two time points spaced at least a year apart, focusing on groups selected for parity and menopausal status: (1) pre-menopausal and nulliparous, (2) pre-menopausal with ≥1 live birth, (3) post-menopausal and nulliparous, and (4) post-menopausal with ≥1 live birth. Study specimens were anonymized, with detailed risk annotation.

Samples for our study were selected based on whether the participant had specimens and data available at least two visits, spaced at least 1 year apart. When more than two longitudinal samples were present, we requested all samples. The time interval between visits was allowed to vary, provided it was greater than 1 year.

### Tissue acquisition and processing

#### Breast tissue

Six core samples were taken from the upper outer quadrant of the breast from each donor under local anesthesia. Within 5 min of procurement, each core was placed into an embedding cassette, and the cassettes were placed into 10% buffered formalin and stored at room temperature. The specimens were then embedded in paraffin. After flash freezing in liquid nitrogen, frozen cores were placed in labeled, chilled cryovials, and stored in liquid nitrogen at −166.2 °C. 84 breast tissue samples, with 50 mg of breast tissue per sample, were shipped to UCLA neurogenetics core laboratory, where DNA and RNA were extracted using the AllPrep kit. 38 of 84 samples had low DNA yield (>20 μl at 100 ng/μl) potentially because of increased percent of fatty tissue and DNA extraction was repeated using leftover tissue from these specimens, with adequate yields in all but one case (0.8 μg total). Extracted DNA was then used for bisulfite sequencing experiments.

#### Peripheral blood specimens

At the Komen Tissue Bank, blood was drawn into the blood collection tube (EDTA 9 ml) and gently mixed. After centrifugation, plasma was withdrawn and the remaining red cells and buffy coat were stored at −80 °C until DNA extraction. DNA was extracted from the blood cells at Indiana CTSI Specimen Storage Facility lab using an AutogenFlex Star (SN 401033) instrument and the Flexigene AGF3000 blood kit for DNA extractions from whole fresh and frozen blood. Four 50 µl aliquots of each sample were pipetted into pre-labeled DNAstable tubes, and stored at ambient temperature. These DNA samples were then shipped to the UCLA for bisulfite sequencing experiments.

### DNA extraction

AllPrep DNA/RNA/miRNA Universal Kit (Qiagen, cat # 80224) was used for the DNA extractions for frozen tissue samples. 30 mg of frozen tissue was lysed with 600 ul guanidine-isothiocyanate-containing Buffer RLT Plus in a 2.0 ml micro centrifuge tube, and homogenized using TissueLyser II (Qiagen) with 5 mm stainless steel beads. Tissue lysate was continued with the AllPrep protocol for simultaneous extraction of genomic DNA and total RNA using RNeasy Mini spin column technology.

### DNA methylation data pre-processing

Bisulfite conversion using the Zymo EZ DNA Methylation Kit (ZymoResearch, Orange, CA, USA) as well as subsequent hybridization of the HumanMethylation450k Bead Chip (Illumina, SanDiego, CA), and scanning (iScan, Illumina) was performed according to the manufacturer′s protocols by applying standard settings. DNA methylation levels (*β* values) were determined by calculating the ratio of intensities between methylated (signal A) and un-methylated (signal B) sites. Specifically, the *β* value was calculated from the intensity of the methylated (M corresponding to signal A) and un-methylated (U corresponding to signal B) sites, as the ratio of fluorescent signals *β* = Max(M,0)/[Max(M,0) + Max(U,0) + 100]. Thus, *β* values range from 0 (completely un-methylated) to 1 (completely methylated).

#### Laboratory performance

We analyzed 22 sets of duplicate samples (13 peripheral blood and 9 breasts) in order to examine for concordance. Blood samples and breast samples were randomized across the Illumina chip to avoid confounding due to technical sources of variation.

### Statistical methods and analysis

#### Study variables

Survey data were available on age at tissue donation, education, ethnicity, age at menarche, menopausal status, age at menopause, gravidity, parity, duration of breastfeeding (months), use of oral contraceptives, and hormone replacement (ever used). We examined the covariate Total Menstrual Years, which was calculated as the difference between age at menopause if post-menopausal (replaced by current age if pre-menopausal) and age at menarche.

#### Epigenetic biomarker of aging

The epigenetic measure of tissue age is calculated by combining the DNA methylation levels of 353 dinucleotide markers known as cytosine phosphate guanines or CpGs [1]. The weighted average of these 353 epigenetic markers gives rise to an estimate of tissue age (in units of years), which is referred to as “DNA methylation age” (DNAm age) or as “epigenetic age.” This epigenetic clock method to estimate age applies to any tissue or cell type that contains DNA (with the exception of sperm) and applies to all 3 versions of the Illumina methylation array (Infinium 27K, Infinium 450K, and the most recent EPIC array). Mathematical details and software tutorials for the epigenetic clock can be found in the Additional files of Horvath [1]. An online age calculator can be found at our webpage (https://dnamage.genetics.ucla.edu).

#### Multivariate analysis

Pearson correlation was examined between DNAm age and chronologic age in both breast and peripheral blood. We used multivariate linear regression models in order to examine relationships between DNAm age and the demographic and reproductive variables listed above. Because DNAm age was available at multiple time points for both breast and peripheral blood, we used linear mixed effects models to examine the association between longitudinal changes in DNAm age and the dependent variables listed above.

#### CpG islands

To delve more deeply into the difference between breast and blood tissue, we looked at the effect of chronological age on the methylation levels of individual CpGs. It is well known that CpGs islands, regions with a high frequency of CpG sites located at or near the transcription start site of genes, tend to gain methylation with age, while those outside of islands tend to lose methylation levels [16]. We calculated mean methylation at CpGs within islands for each participant and compared these values to those located in other chromosomal locations, with separate analyses for breast and peripheral blood. Pearson correlation statistic of methylation levels against age was calculated for breast and peripheral blood. Non-parametric group comparison tests (Kruskal–Wallis) were performed to test for mean differences in methylation between (1) island versus non-island CpGs, and (2) breast versus peripheral blood groups.

## Results

### Comparison of epigenetic clock in breast and peripheral blood samples from healthy women donors

We received DNA isolated from peripheral blood and matched breast tissue samples from 49 healthy women who donated at two or more time points. 9 of the women in our sample did not have adequate breast tissue specimens and our analysis was focused on the remaining 40 women who had matched peripheral blood and breast samples. Individuals in our sample were aged 18–65 years, with 22 pre-menopausal, 18 post-menopausal, 17 nulliparous, and 23 with at least one live birth. We analyzed data from *n* = 192 breast and peripheral blood samples from 2 or more time points from 40 individuals. All samples were profiled in a single batch using the Illumina 450k platform. 24 of these samples were duplicates (13 peripheral blood, 9 breast). Analysis of 24 sets of duplicate samples revealed excellent concordance (correlation coefficient 0.97, *p* < 0.0001) between samples. Blood samples and breast samples were randomized across the Illumina chip to avoid confounding due to technical sources of variation.

An overview of our participant population is presented in Table 1. Chronological age at the time of sample collection ranged from 18 to 65 years (mean age = 42.5) at the first time point, 21–70 years (mean age = 46.7) at the second time point, with a mean age difference between the two assessments of 4.2 years (range 2–7 years) between the two assessments. There were two individuals who were pre-menopausal at the first visit and post-menopausal at the second. There were three individuals who were nulliparous at the first visit and had at least 1 live birth at the second.

**Table 1:** Overview of the KTB breast and peripheral blood methylation dataset

	Age < 50 years	Age ≥ 50 years	Overall
Participants (*n*)	24	16	40
Age at first donation (mean, SD)	33.8 (8.8)	55.4 (4.3)	42.5 (12.9)
Years between first and second donation (mean, range)	3.9 (2–7)	4.7 (3–6)	4.2 (2–7)
Nulliparous (*n*)	12	5	17
No.of live births (mean, SD)	0.92 (1.0)	2.0 (1.4)	1.2 (1.2)
No. of pregnancies (mean, SD)	1.0 (1.3)	2.1 (1.5)	1.5 (1.5)
Pre-menopausal (*n*)	20	2	22
Total menstrual years (mean, range)	20.2 (5–37)	32.6 (13–41)	25.3 (5–41)
Age at menopause (mean, SD)	33.0 (7.2)	45.1 (6.3)	42.5 (8.0)

Participant characteristics for baseline demographic and reproductive variables

Our sample included 6 Hispanic, 1 African American, and 1 East Asian woman. Two individuals were Ashkenazi Jewish. We have previously shown that race/ethnicity has a weak association with epigenetic age acceleration in blood tissue [17]. However, race/ethnicity was not associated with epigenetic age acceleration in this study, which might reflect the low sample sizes.

**Fig. 1:**
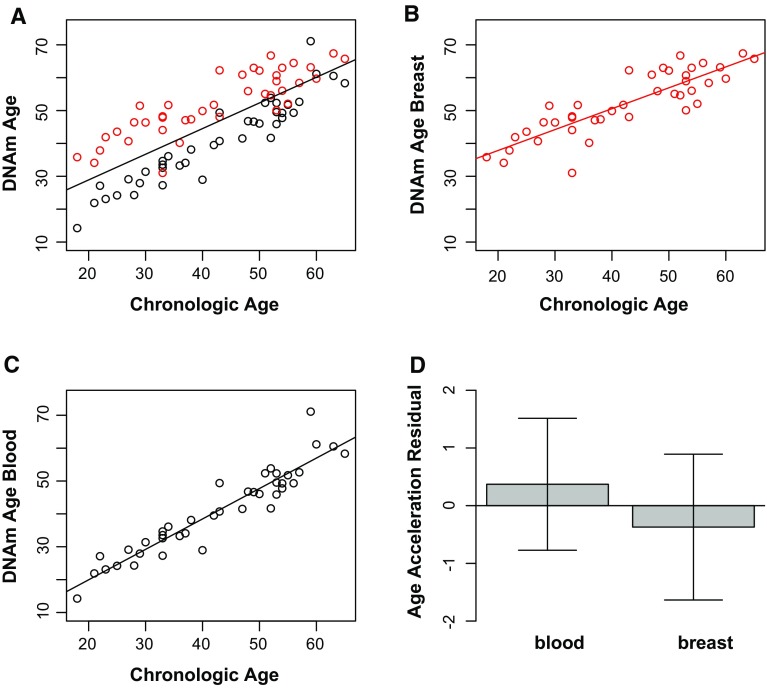
Epigenetic clock analysis of breast tissue and peripheral blood. **a** Scatter plot of DNAm age (*y*-axis) versus chronological age (*x*-axis) for breast tissue (*red*) and peripheral blood (*black*) samples at baseline visit, correlation coefficient = 0.8, *p* value = 5.5 × 10^−19^. Regression lines demonstrate the linear relationship between DNAm age and chronologic age in all samples (**a**), in breast (**b**, *ρ* = 0.86, *p* value = 1.2 × 10^−12^) and in blood (**c**, *ρ* = 0.94, *p* value = 2.4 × 10^−19^) separately. **d** This bar plot shows the relationship between epigenetic age acceleration and tissue type, using data at baseline visit, demonstrating the mean value (*y*-axis) and one standard error, with *p* value = 1.6 × 10^−11^ from the results from a non-parametric group comparison test (Kruskal–Wallis)

As expected, DNAm age has a strong linear relationship with chronological age in all samples, with the correlation coefficient being higher for peripheral blood (*ρ* = 0.94, *p* < 0.0001 at the baseline visit) than for breast tissue (*ρ* = 0.86, *p* < 0.0001) (Fig. 1, panels A–C). Using the Fisher *r*-to-*z* transformation, a *z*-value of 1.74 was calculated with a one-tailed *p* value of 0.04 revealing a significant difference between the correlation coefficients of 0.94 and 0.86. To formally measure age acceleration effects, we defined age acceleration residual for each sample by taking the residuals from the linear regression of DNAm age on chronologic age. This measure was significantly higher in breast compared with blood (Fig. 1d).

**Fig. 2:**
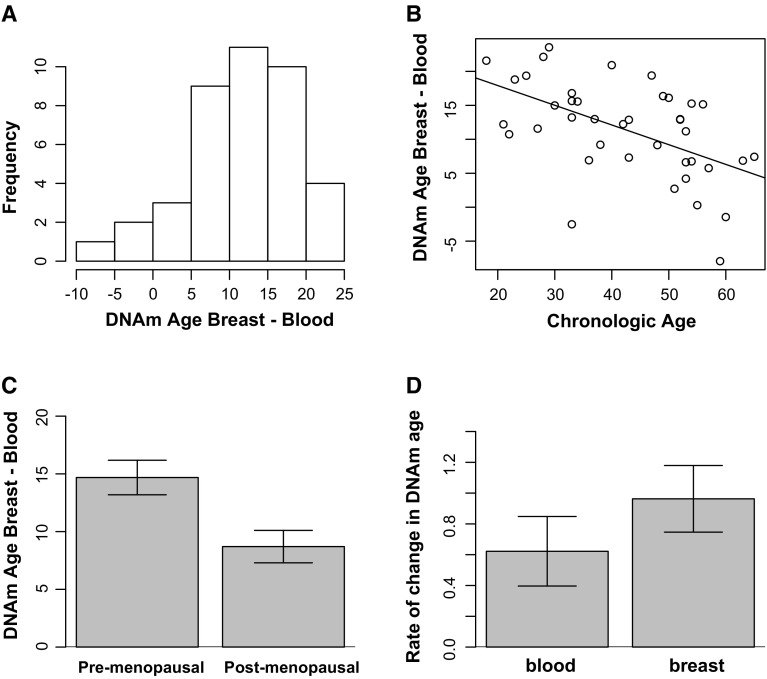
Tissue difference in DNAm age between breast and blood. **a** Frequency distribution of the absolute difference DNAm age breast and DNAm age blood at first visit. On average, DNAm age of breast is 11.4 years older than that of blood (SD = 7.1, range −7.9 to 23.5 years). **b** While DNAm age of breast is significantly higher than blood at younger ages, the gap closes with advancing age. This scatter plot shows a strong inverse correlation between chronologic age and the absolute difference DNAm age breast and DNAm age blood (correlation coefficient = −0.53, *p* = 4.4 × 10^−4^), **c** The absolute difference between DNAm age breast and DNAm age blood is higher in pre-menopausal women (mean 13.9, SD 6.3 years) compared with post-menopausal women (mean 8.3, SD 6.9 years, with *p* value = 0.0098 from non-parametric group comparison Kruskal–Wallis testing). **d** The rate of change in DNAm age is higher in blood than breast tissue. This bar plot shows the relationship between rate of change in DNAm age and tissue type, demonstrating the mean value (*y*-axis) and one standard error. While the DNAm age in breast starts out higher, DNAm age in blood eventually catches up to breast tissue, with results from a non-parametric group comparison test reveal a significantly faster rise in DNAm age in blood than breast over time, with *p* value = 0.038 (Kruskal–Wallis)

When we examine the linear relationship between DNAm age and chronologic age in breast and blood tissue separately, we can calculate the distance between these two regression lines to compare the difference in DNAm age between breast and blood for varying chronologic ages. For women aged 21 years, breast appears 17.5 years older than blood, whereas for women aged 55 years, breast appears 8 years older than blood. The full distribution of tissue differences of DNAm age between breast and blood is shown in Fig. 2a. For the majority of participants, breast was much older than blood. Interestingly, the degree of separation in DNAm age between breast and blood is greatest at younger ages, and there is convergence around the age range that is typically associated with the menopausal transition. The absolute difference in DNAm age between breast and blood was inversely correlated with advancing age (*r* = −0.53, *p* = 4.4 × 10^−4^) (Fig. 2b). We found that the difference between breast and blood was significantly higher in pre-menopausal women than in post-menopausal women (*p* = 0.0098 using the non-parametric Kruskal–Wallis test) (Fig. 2c). However, in multivariate analyses, we found that there was not a significant association between difference in DNAm age between breast and blood and menopausal status (*β* = 1.1 for pre-menopausal women, *p* = 0.68) after adjusting for chronologic age (*β* = −0.26, *p* = 0.02).

### Longitudinal changes in DNAm age with advancing chronologic age

**Fig. 3:**
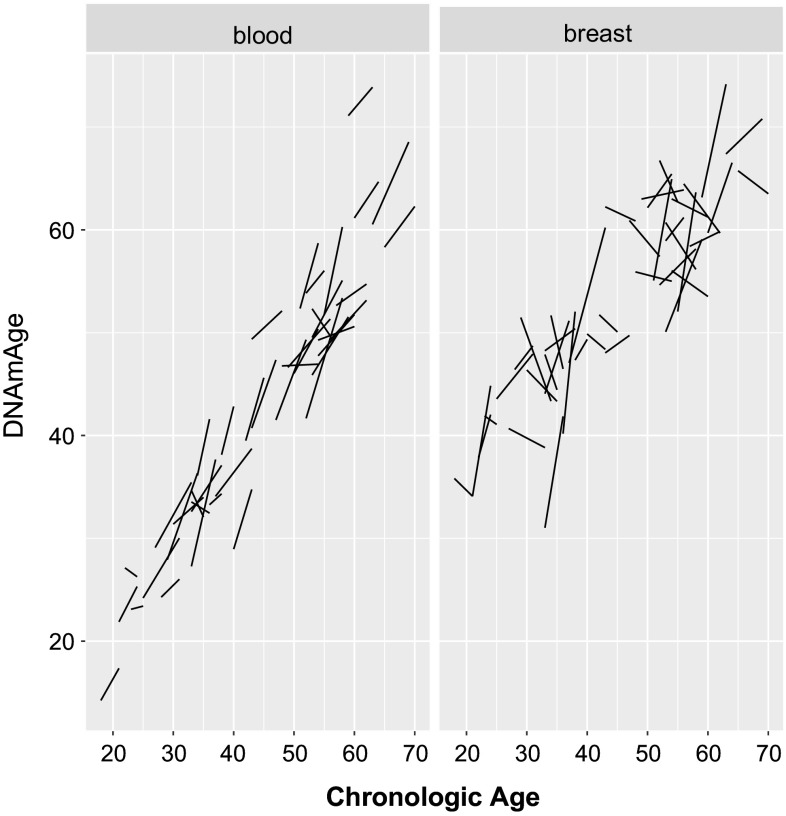
Individual trajectories of DNAm age with advancing chronologic age. This plot shows the longitudinal changes in DNAm age of blood (*left panel*) and breast (*right panel*) for each individual at visits 1 and 2

Figure 3 shows individual trajectories of DNAm age over the study interval in both breast tissue and blood. To examine longitudinal changes in DNAm age over time between breast tissue and blood, we defined the rate of change in DNAm age as follows:

$${\text{Rate}}\,{\text{of}}\,{\text{change}}\,{\text{in}}\,{\text{DNAm}}\,{\text{age}} = \frac{{({\text{DNAm}}\,{\text{age}}\,{\text{visit}}\,2 - {\text{DNAm}}\,{\text{age}}\,{\text{visit}}\,1)}}{{({\text{Age}}\,{\text{visit}}\,2 - {\text{Age}}\,{\text{visit}}\,1)}}.$$

We can examine the full distribution of rate of change in DNAm age in both breast and blood (Supplementary Fig. 1a, b). We find that the rate of change in DNAm age is significantly higher in blood (mean = 0.97) than breast (mean = 0.61) (*p* = 0.038 using the non-parametric comparison Kruskal–Wallis test) in our sample (Fig. 2d).

While chronological age differences between the first and second visits ranged from 2 to 7 years (mean 4.2 years, SD = 1.5), the biologic age difference ranged from −2.6 to 11.7 years (mean 4.1 years, SD = 3.2) as measured by peripheral blood, and the biologic age difference in breast tissue ranged from −8.1 to 13.1 years (mean 2.0 years, SD 5.6). It is of note that while 4 peripheral blood samples demonstrated a younger biologic age at visit 2 than visit 1, a greater number of breast samples (*N* = 18) had an estimated DNAm age that was younger at visit 2 than visit 1. In only one woman, both breast tissue and blood had estimated younger biologic ages at follow-up compared with baseline. *T*-tests comparing the intraindividual differences in age between visits 1 and 2 revealed no significant difference between change in DNAm age of blood and change in chronologic age across visits (mean change of 4.1 years vs. 4.2 years *p* = 0.81), while there was a significant difference between change in DNAm age of breast and change in chronologic age across visits (mean change of 2.0 years vs. 4.2 years, *p* = 0.025).

Using a linear mixed effects model, we find that the difference between DNAm age of breast and blood is associated with chronologic age (*β* = −0.40, 95% CI −0.26 to −0.54), and not associated with parity (*β* = −2.1 for nulliparous women, 95% CI −0.78 to 5.0) or menstrual status (*β* = 0.34, 95% CI −3.4 to 4.0). There was a borderline association between the difference between DNAm age of breast and blood and total menstrual years (*β* = 0.16, 95% CI −0.02 to 0.35).

### Age effects on individual CpGs differ between breast and blood

**Fig. 4:**
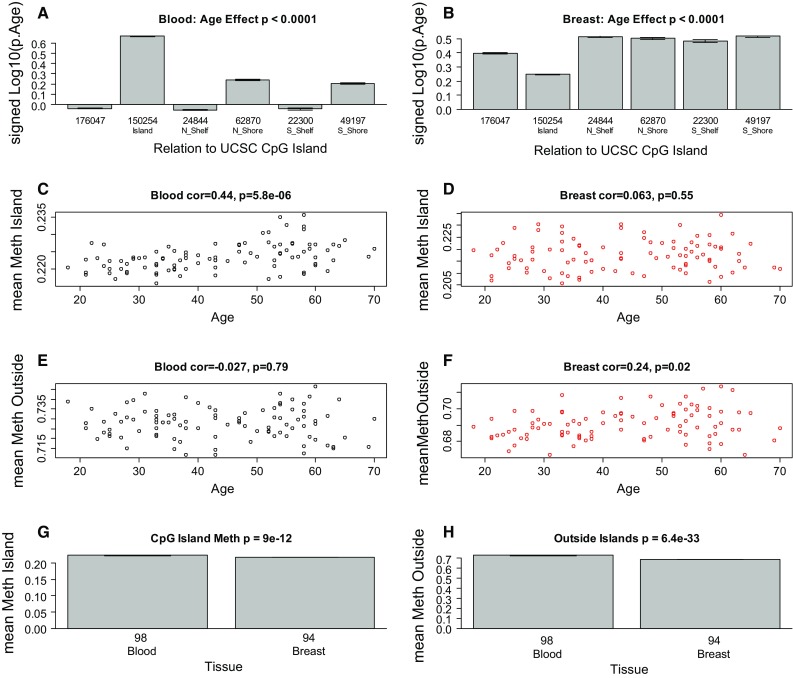
DNA methylation levels inside and outside of CpG islands. **a** Effect of chronological age on CpG methylation versus chromosomal location in **a** blood and **b** breast tissue. The *y*-axis of the bar plot shows the log (base 10)-transformed correlation test *p* value (whose sign reflects the sign of the correlation). **c**, **d** Mean methylation levels in CpG islands versus chronological age in **c** blood and **d** breast tissue. **e**, **f** Mean methylation levels outside of CpG islands versus chronological age in **e** blood and **f** breast tissue. **g** Mean methylation in CpG islands versus tissue type. **h** Mean methylation outside of CpG islands versus tissue type

We also studied the effects of chronological age on individual CpGs in our dataset. Aging effects were quite different between breast and blood tissue (Fig. 4). In blood, CpGs that are located in CpG islands tend to have a positive correlation with chronological age (Fig. 4a). This is a well-known effect, which has been observed in many other tissues [16, 18, 19], and is also reflected by a positive correlation between chronological age and the average methylation level of island CpGs in blood (*ρ* = 0.44, *p* = 5.8 × 10^−6^, Fig. 4c). Surprisingly, this well-known result from blood cannot be reproduced in breast tissue (Fig. 4b, d). It is even more surprising that the average DNA methylation levels of CpGs that are located outside of CpG islands exhibit a positive correlation with chronological age in breast tissue (*ρ* = 0.24, *p* = 0.02, Fig. 4f), which contrasts with the non-significant (negative) association in blood tissue (Fig. 4e). Overall, these results suggest that aging effects differ greatly between the two tissues. We find that the mean methylation levels of CpGs are lower in breast tissue samples (compared with blood samples). The effect is particularly significant for CpGs located outside of CpG islands (*p* = 6.4 × 10^−33^, Fig. 4h) but can even be observed for CpGs located inside of CpG islands (*p* = 9 × 10^−12^, Fig. 4g).

## Discussion

To our knowledge, this is the first study to demonstrate that breast tissue epigenetic age exceeds that of blood tissue in healthy female donors. In addition to validating our earlier finding of age elevation in breast tissue, we further demonstrate that the magnitude of the difference between epigenetic age of breast and blood is highest in the youngest women in our study (age 20–30 years) and gradually diminishes with advancing age. As women approach the age of the menopausal transition, we found that the epigenetic of age of blood approaches that of the breast.

Our studies were performed on whole breast tissue. Diverse types of cells make up whole breast tissue, with the majority of cells being adipocytes. Other types of cells include epithelial cells, cuboidal cells, myoepithelial cells, fibroblasts, inflammatory cells, vascular endothelial cells, preadipocytes, and adipose tissue macrophages. This raises the possibility that the magnitude of the effects we observe, of breast tissue DNAm age being greater than other tissues, might be an underestimation, since it is possible that not all of the cells of the heterogenous sample have experienced this effect. Since it is difficult to extract DNA from adipose tissue, we suspect that the majority of DNA extracted from our whole breast tissues was from epithelial and myoepithelial cells. We hypothesize that the myoepithelial and epithelial cells in the mammary gland are the cells responsible for the observed increase in DNAm age, because these cells are exposed to variability in hormone levels including estrogen and growth hormone during puberty, development, and menstrual cycles, and oxytocin during lactation, with resultant proliferation and cell cycling. It is of note that across most cell types and tissues (except breast), there is excellent agreement in estimated DNAm age from different cells and tissues. Further experiments are needed to confirm that DNAm age of isolated breast epithelial and myoepithelial cells is higher than observed in our study. Furthermore, with advancing age, the composition of the types of cells within the breast changes, with increasing proportion of adipose tissue. Therefore, it is unclear whether the deceleration observed in our study as a woman approaches menopause is a true deceleration or whether this observation is a result of loss of the cell types that experience the deceleration.

We found that the biologic age of peripheral blood was tightly correlated with chronologic age in the age range that we studied, consistent with previous studies. The degree of intraindividual and interindividual variability in DNAm age of healthy breast tissue had not been previously quantified. At the baseline visit, the variance in chronologic age was 167.7, with the variance in biologic age as measured by DNAm age of the peripheral blood being 161.8, and breast 92.7. Examining the intraindividual difference in DNAm age between visits 1 and 2, we note that the variance of this difference is greater in breast (31.4) compared with blood (10.4) and chronologic age (2.3). While we note that a higher proportion of women (18/40) appeared to have “younger” breasts at follow-up, than the proportion of women (2/40) who appeared to have “younger” blood at follow-up, there was only one woman who had both younger blood and breast tissue, suggesting that this observation may be attributable to the variability in this measurement. Further studies are needed to examine whether true reversal of epigenetic aging in breast tissue ever occurs under any circumstances.

We have observed a strong linear relationship between DNAm age and chronologic age for both breast and blood tissue. We infer that a non-linear increase in DNAm age of breast occurs prior to the adulthood as evidenced by the higher DNAm age of breast at the youngest adult ages we have studied. We note that the rate of change in breast tissue is slower than that of both chronologic age and the rate of change in peripheral blood. Further studies may examine for non-linear effects around the age of the menopausal transition, and for the relationship between non-linear change in DNAm age and the risk of later developing breast cancer.

Our findings raise the question of what factors regulate the aging process in the breast, and whether there are common mechanisms underlying accelerated aging and carcinogenesis. We postulate that biologic aging in female breast tissue may be accelerated beginning at puberty, and may be linked to stimulation by estrogen, progesterone, and oxytocin, and cell cycling, so that by early adulthood the DNAm age in female breast is higher than that of other tissues, as we observe. Breast cancer overall shows a bimodal distribution with respect to age, with the first peak occurring at age 50 years (more commonly medullary or inflammatory breast cancer), and the second peak reached at over 70 years of age (more commonly estrogen and progesterone receptor-positive lobular or mucinous cancers) [20]. It is well known that risk of breast cancer is linked with endogenous estrogen and progesterone exposure, including early menarche, late menopause, nulliparity, age at first birth [21–25], as well as exogenous exposure to hormone replacement [26]. These relationships are true in breast cancers diagnosed in pre-menopausal as well as post-menopausal women [27], and in breast cancers that are estrogen receptor-positive or negative [28]. Estrogen is known to be involved in the development of the mammary gland and epithelial stem cell regulation [29–31]. Estrogen regulates cell cycle progression through the cyclin-dependent kinase pathway [32]. Proliferation rates of normal breast epithelial cells as measured by labeling index studies have shown a decline in proliferation with advancing age [33–35]. However, the relationship between estrogen and progesterone stimulation, chronic cell cycling, and cellular senescence in breast tissue is poorly understood.

Previous studies have examined the relationship of hormonal risk factors and the age incidence of breast cancer [35, 36]. Noting the linear log–log relationship between cancer incidence and age is not observed in breast cancer compared with most other non-hormone-dependent cancers, Pike et al. constructed a mathematical model describing the relationship between cancer incidence and age that incorporated a decline in the rate of breast tissue aging starting at age 40 and ending at the last menstrual period. This modification led to excellent agreement between observed and predicted effects of age at menarche, first full-term pregnancy, and menopause on breast cancer risk. The results of their modeling suggest that hormones have a major role in determining breast tissue age, and that the important etiologic elements for breast cancer appear to be present in pre-menopausal women and sharply reduced following the menopause [35, 36]. Our finding that epigenetic age elevation in adult female breast is highest following development and gradually diminishes with advancing age and the menopausal transition is consistent with the concept that hormonal factors drive breast aging and the degree of difference between breast and other tissues is reduced with advancing age.

Many reproductive factors may influence changes in DNAm age of healthy female breast tissue. While our explorative, hypothesis-generating study reported here was not powered to answer these important questions, the Komen Tissue Bank provides a rich resource to examine the influence of cumulative estrogen exposure on DNAm age. Future studies are needed to examine overlapping effects of nulliparity, age at first birth minus age at menarche, age at menopause, exogenous estrogen exposure, lactation, fertility treatments, and proximity of pregnancy to the time of sampling, and whether these factors modulate DNAm age acceleration. Using large cohort studies, we have recently shown that early menopause is associated with accelerated epigenetic age of blood [14]. Our limited sample size did not allow us to test whether a similar age acceleration effect can also be observed in breast tissue from post-menopausal women. We hypothesize an acceleration in breast tissue epigenetic aging occurs beginning a puberty, leading to an elevated DNAm age that persists until menopause. We anticipate that the rate of epigenetic aging following the menopausal transition may coincide with the rate of other tissues. Furthermore, with advancing age, the composition of the types of cells within the breast changes, with increasing proportion of adipose tissue. Therefore, it is unclear whether the deceleration observed in our study as a woman approaches menopause is a true deceleration or whether this observation is a result of loss of the cell types that experience the deceleration. We will direct future studies in healthy women to examine accelerations in epigenetic aging, cumulative hormone exposure, and cellular changes in response to pregnancy. Future studies should also explore epigenetic age differences in male and female breast tissue, and examine whether the degree of epigenetic age acceleration is associated with increased risk of breast cancer. In the present study, we confirm that healthy breast tissue exhibits accelerated aging relative to other tissues, and we have previously shown that epigenetic age of normal adjacent breast tissue in breast cancer patients is accelerated. While the current study does not directly compare methylation patterns in healthy breast tissue versus normal adjacent breast tissue in breast cancer patients, future studies are needed to assess for cancer field effects on epigenetic aging.

Further analyses will be improved by the addition of variables related to breast cancer risk, including history of benign breast disease, and family history. While our study was not powered to examine these effects, future studies will include these variables and test whether they are associated with accelerations in breast epigenetic age. By extending the Pike model to data from the Nurses’ Health Study, a large cohort study examining risk factors for major chronic diseases in women, Rosner and Colditz were able to show that age at all births, benign breast disease, type of menopause and use of post-menopausal hormones, and alcohol use have long-term influence on breast cancer incidence [37, 38]. Furthermore, recent results reported using survey data from The Growing Up Today Study, a large study of girls who are daughters of Nurses’ Health Study participants, show body size factors from pregnancy to late adolescence were associated with risk of benign breast disease [39]. Future work should be directed towards the important question of whether epigenetic age of the breast is related to body size, body mass index, and risk of benign breast disease.

Mammographic breast density is known to be associated with breast cancer risk [40–42]. A longitudinal cohort study tracking change in percent mammographic density over 3–12 years revealed a slowing in the rate of decline, with annual rates of decline of 1.4, 0.7, and 0.1% at age 50, 57, and 60 years, respectively [43]. Higher body mass index, greater parity, and younger age at first child’s birth are associated with lower percent mammographic density, while more immediate factors such as the use of hormone therapy and the menopausal transition affect the rate of change in mammographic breast density [43, 44]. Our work also motivates the question of whether there may be a link between epigenetic age and breast density, whether accelerated breast epigenetic aging may explain the difference in biology and risk factors for pre-menopausal and post-menopausal breast cancer, and whether epigenetic signatures in female breast biopsies may provide additional information, beyond known risk factors, of breast cancer risk in a population of women at high risk for breast cancer. Identifying mechanisms by which age-related changes may contribute to breast cancer development and progression may aid in the development of a clinical biomarker of elevated breast cancer risk.

## Electronic supplementary material

Below is the link to the electronic supplementary material.

Supplementary material 1 (PDF 176 kb). Supplemental Fig. 1. Rate of change in DNAm age with advancing chronologic age differs by tissue type. A) Frequency distribution of rate of change in DNAm age in breast (left panel) and blood (right panel).
