# Supplementary figures and images for: DNA methylation age is elevated in breast tissue of healthy women
Source: Breast Cancer Res Treat. 2017 Mar 31;164(1):209–19. doi: 10.1007/s10549-017-4218-4 (PMC5487725; doi:10.1007/s10549-017-4218-4)

**Breast**

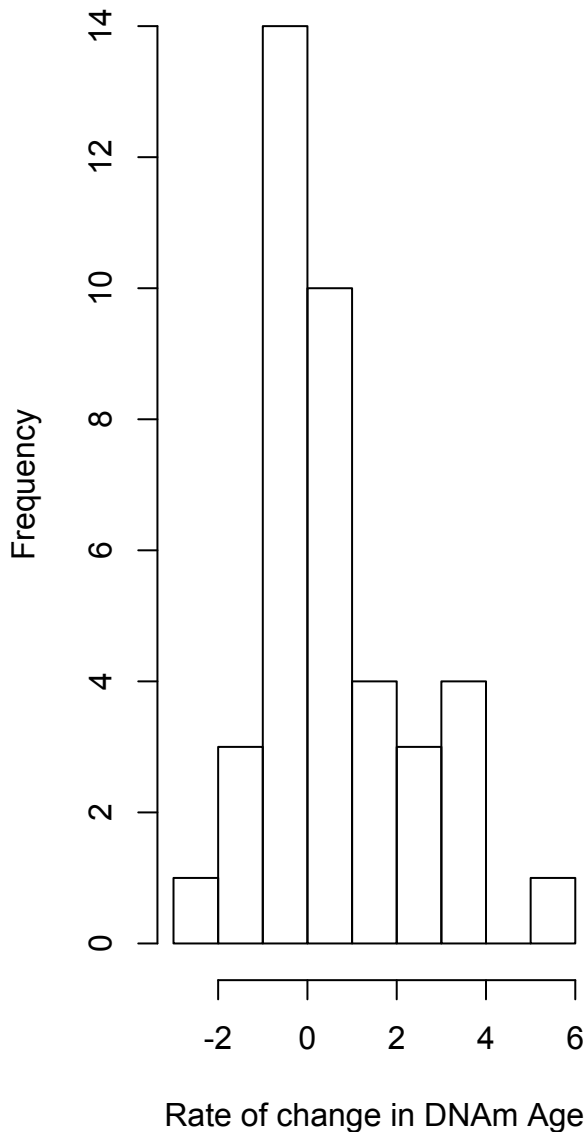

**Blood**

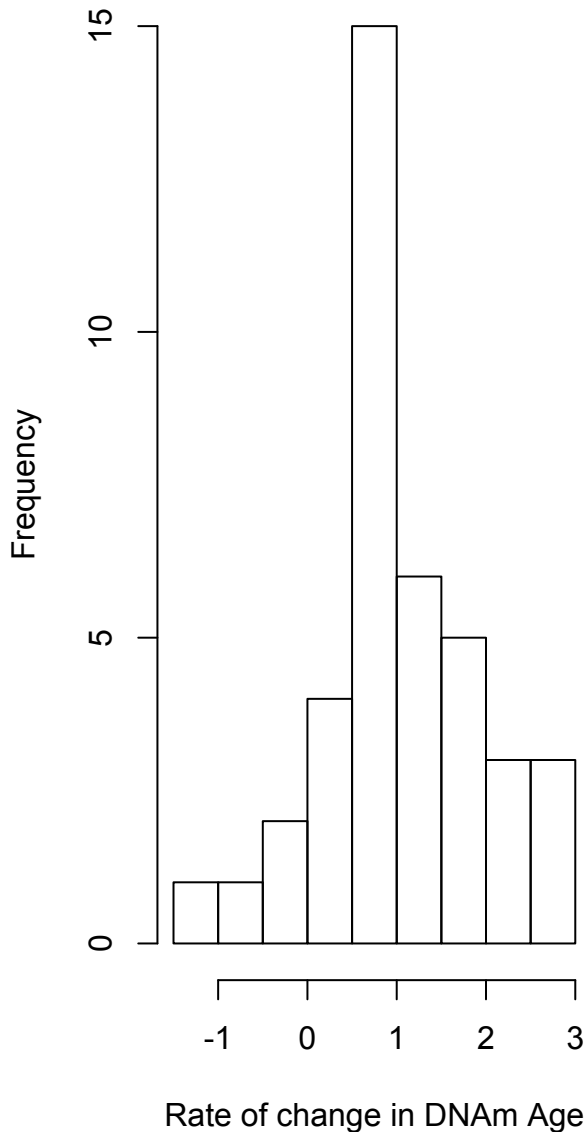

Supplement: Supplementary file 1 — Supplementary material 1 (PDF 176 kb). Supplemental Fig. 1. Rate of change in DNAm age with advancing chronologic age differs by tissue type. A) Frequency distribution of rate of change in DNAm age in breast (left panel) and blood (right panel). [file 10549_2017_4218_MOESM1_ESM.pdf]
